# Addressing the elephant in the room: understanding functional neurological symptom disorder through the lens of culture and religion

**DOI:** 10.3389/fneur.2023.1174364

**Published:** 2023-08-31

**Authors:** Iram Zehra Bokharey, Urusa Fahim, Khola Tahir, Zarish Shireen

**Affiliations:** ^1^Department of Psychiatry and Behavioral Sciences, Mayo Hospital, Lahore, Pakistan; ^2^Department of Business Administration, Kinnaird College for Women, Lahore, Pakistan; ^3^Department of Psychology, Forman Christian College (A Chartered University), Lahore, Pakistan

**Keywords:** functional neurological symptom disorder, thematic analysis, cultural and religious values, romantic relationships, sexual suppression

## Abstract

Owing to the dearth of scholarly works to understand the presence of Functional Neurological Symptom Disorder (FNSD) among mental health patients in Pakistan, this study sought to understand how cultural and religious conflicts are implicated in the aetiology of FNSD. The study recruited 22 participants, comprising five men and 17 women. The participants were recruited from the Department of Psychiatry at Services Hospital, Lahore, Pakistan. Semi-structured interviews were conducted and analyzed through Thematic Analysis. The two main themes identified in this study were cultural and religious values and beliefs about romantic relationships. Within the cultural and religious values theme, subthemes of self-perception, a conviction in religious beliefs, and sexual suppression were identified. Furthermore, the subthemes of beliefs about romantic relationships were family’s approval, engagement against wishes, and fear of exposure. The two main themes are interconnected: beliefs about romantic relationships were interpreted and experienced through the perspective of religion and culture. To summarize, this study concluded that stressors related to culture and religion are significant contributing factors in the development of FNSD. This study has important implications for mental health professionals, as awareness around the interplay of cultural as well as religious beliefs and FNSD will enable them to devise effective and holistic therapeutic intervention.

## Introduction

Functional Neurological Symptom Disorder (FNSD) or Conversion Disorder is a psychiatric disorder in which the symptoms affect voluntary motor and sensory functionality that cannot be explained by a neurological or general medical condition (1). Common symptoms of FNSD include blindness, paralysis, dystonia, psychogenic non-epileptic seizures, swallowing difficulties, motor tics, and difficulty walking (2). The prevalence rate of this disorder varies widely across different cultures. A recent study reported that one in five individuals visiting outpatient psychiatric departments has medically unexplained neurological symptoms (3). According to American Psychiatric Association (4), the estimated yearly prevalence of FNSD is 4–12 cases per 100,000 people. In South Asian countries such as Pakistan, India, and Bangladesh, the prevalence is as high as 31% and twice as common in women than in men (5, 6).

Despite psychodynamic, biological, behavioral, cognitive and socio-cultural theories’ attempts to explain the causal factors of FNSD, its aetiology remains an enigma (7). Each theory explains the development of FNSD relevant to its own understanding. However, an integrated biopsychosocial model of FNSD emphasizes that the interpretation of the physical symptoms experienced by individuals with FNSD are highly influenced by personal experiences, observation of others and socio-cultural factors (2). As the prevalence of FNSD tends to be significantly different in industrialized and developing countries (8), it is assumed that socio-cultural factors tend to play a significant role in the development of FNSD. Thus, FNSD is more common in cultures where direct and honest expression of desires and emotions is discouraged and frowned upon (9).

On the contrary, conformity to religious and cultural norms is deemed virtuous and acceptable. In such cultures, FNSD becomes a strategy for expressing suppressed emotions and desires (5). Furthermore, the prevalence of FNSD is higher among individuals exhibiting higher degree of religiosity, lower levels of literacy and socioeconomic status (1), those who reside in rural areas, individuals of younger ages and women (6). It is also common where physical symptoms are given more importance than psychological symptoms due to lack of psychological sophistication (10). Developmental psychologists propose that adults transmit cultural socialization to the younger generation and that the learned behaviors are internalized and exhibited for certain benefits. This phenomenon may give rise to developmental trajectories that could ultimately contribute toward the onset of FNSD in adults (11).

South Asia is a primary example of an environment where all of the factors mentioned above are widely prevalent in a society deeply tied to cultural and religious traditions and with the low human development of the masses. In particular, Pakistan is a developing country where most people belong to lower socioeconomic status (12) and have a low literacy rate. Apart from these factors, being Muslims, people belonging to Pakistan share a set of cultural and religious beliefs which create their moral compass around culture and religion. The ideal Islamic and cultural teachings include, but are not limited to, respect for elders (13), submissiveness to one’s spouse (especially women respecting their husbands), not being vocal about one’s desires and needs, refraining from having intimate relationships outside of marriage, masturbation, and watching porn (14).

Thus, if one wants to explain the development of FNSD in a country like Pakistan, it is imperative to understand the ambivalence or conflict between the “should self” and “want self” (15). Furthermore, the socio-cultural approach toward FNSD has also emphasized the external environment and the patients’ complex, often unconscious, intra-psychic conflicts. The socio-cultural approach accepts the fundamental idea of the psychodynamic perspective that people with FNSD often experience “forbidden” ideas which are not acceptable in their culture or religion (16, 17). This causes turmoil in one’s mind and disturbs the mental state. If these conflicts are not addressed, they are likely to find an expression in somatic symptoms, as in FNSD (5). Therefore, to understand the complex web of dissonance and ambivalence among patients diagnosed with FNSD, this study aimed to understand the impact of cultural and religious beliefs on the onset of FNSD.

## Theoretical framework

Leon Festinger’s theory of Cognitive Dissonance (18) proposed that human beings try to maintain harmony and consistency between their beliefs, attitudes, and behaviors. Cognitive dissonance is created when there is a contradiction between beliefs, attitudes, and behaviors. FNSD is a complex phenomenon. Hence, for a thorough understanding of the subject, i.e., FNSD, it is better to lay out as many details as possible to get a rich perspective. Furthermore, studying FNSD through the dimension of Festinger’s theory of Cognitive Dissonance assumes a vital position when we seek to understand the ambivalence and dissonance inherent in it (19). To better understand the process by which dissonance is created and resolved, Harmon-Jones and Mills (20) suggested examining forms of dissonance using the concept of paradigms from Festinger’s theory. The following paradigms explain the types of dissonance in the accounts narrated by the participants: free-choice paradigm, effort-justification paradigm, belief-disconfirmation paradigm, induced-compliance paradigm, and induced-hypocrisy paradigm are some of the ways to induce cognitive dissonance. In the Free-choice paradigm, people choose a problematic option when presented with evenly attractive and unattractive choices (20).

An example is when a person has to choose between a job that offers stability and starting a business. The latter choice involves some risks. Once the choice has been made, dissonance is likely to occur because of the highlighted negatives of the accepted choice and the positives of the rejected choice (21).

When an individual voluntarily indulges in an unpleasant activity or behavior to achieve the desired outcome, they fit in the effort-justification paradigm (22). For instance, South Asian families spend and show extravagance on weddings to gain social approval. The reason for such behavior is usually attributed to the expectations the family, community or society have of them at the time of an offspring’s wedding.

In the belief-disconfirmation paradigm, individuals only accept the evidence supporting their beliefs and reject the information that does not align with the previous belief system (20, 23). For example, a family on an island amidst a tsunami believes that it will not hit their house because they are spiritual and when their house miraculously evades the tsunami. The disconfirmation further solidifies their belief that their religiosity helped them escape the tsunami.

When a person likes to share information with those with the same beliefs, they act out the belief-disconfirmation paradigm. Festinger’s most ubiquitous concept is the induced-compliance paradigm, which occurs when there is a contradiction between the initial set of beliefs or behaviors and current cognitive patterns or actions (24–26). For instance, an individual engaging in premarital relationships could experience dissonance due to the previously held belief that premarital relationships are wrong.

**Figure 1:**
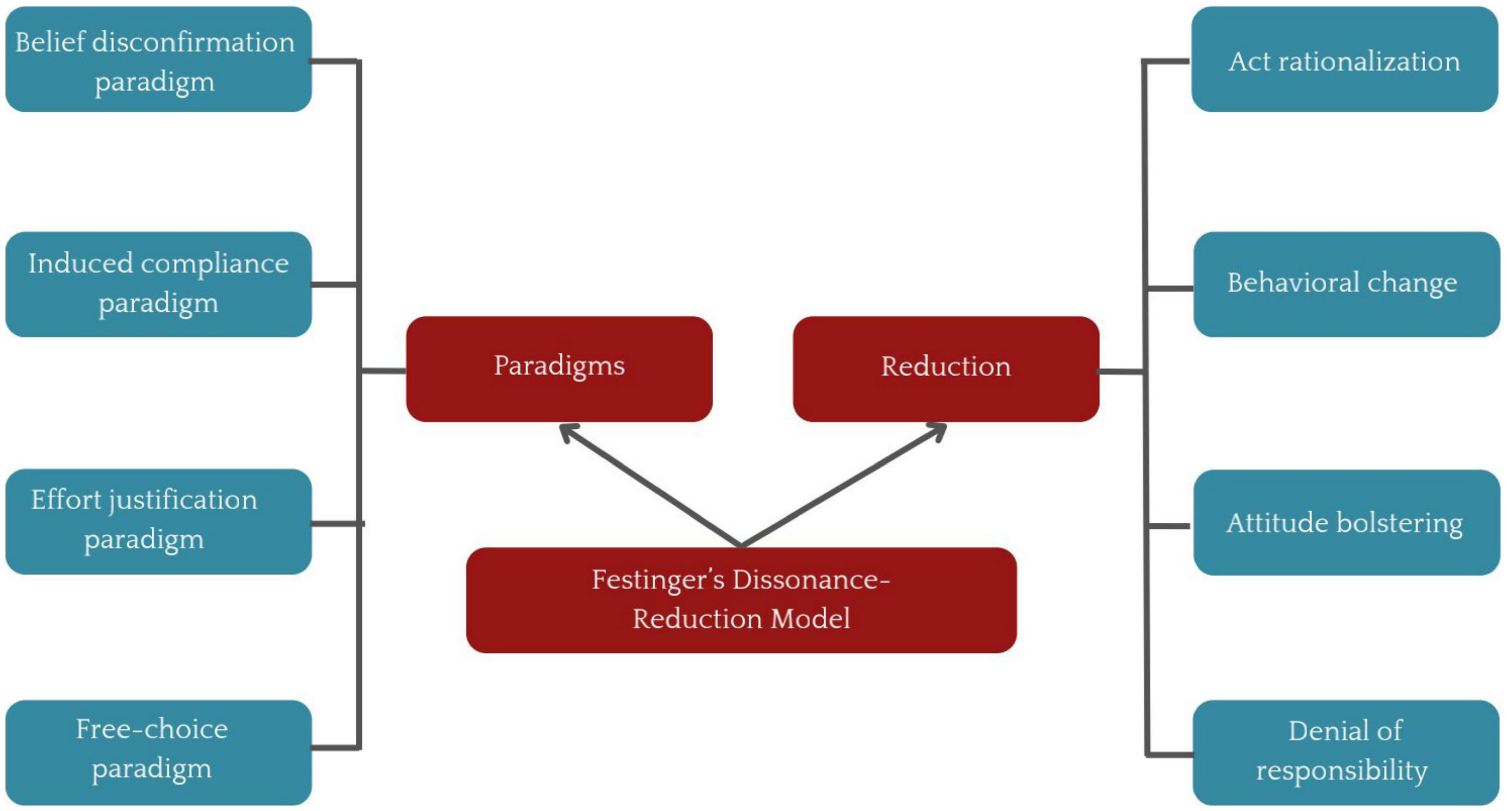
The dissonance-reduction and model given by Leon Festinger.

Individuals try to reduce their cognitive dissonance to align their cognitions with their behaviors through a phenomenon called reduction. The reduction can be made in many ways. For instance, Stangor (27) and McGrath (28) described that an individual reduces the enormity or cognitive dissonance by altering the cognition and bringing changes in one’s behavior to fit the cognition (Behavioral change) (29). Justifying cognition by altering the conflicting behavior is called act rationalization (28, 30). The third form of reduction is attitude bolstering, which justifies cognition by adding new behavior (29, 31). Disagreeing with the information that contradicts the existing perspectives is called Denial of responsibility [(32, 33) as cited in (28, 29)]. Some strategies reduce dissonance temporarily others result in chronic dissonance (Figure 1). Hoshino-Browne et al. (34) proposed that almost all the individuals have one or more of the dissonance reduction strategies, and that these strategies are often culturally driven. The idea behind this concept is that people often use rationalization based on their cultural beliefs. Hence, it can be concluded that cognitive dissonance and its reduction strategies are highly influenced by culture (34, 35). Our mind and body are interdependent; thus, chronic dissonance is likely to cause various mental health problems such as anxiety disorders, depression, and somatoform disorders (36), which can also develop into chronic physical illness.

Despite the high prevalence of FNSD in Pakistan, the existing scholarly accounts still need to address the issue adequately. Existing research has yet to focus on factors such as culture and religion in its development and maintenance. This study was designed to fill this critical gap in the literature by unveiling how cultural and religious factors contribute toward the development and maintenance of FNSD.

## Method

The study employed a qualitative method to comprehensively understand the impact of cultural and religious factors on FNSD. The very nature of the inquiry, which required that the participants felt comfortable while talking about these sensitive topics, seemed best suited to be situated in the qualitative paradigm. Conversely, the quantitative forms of empiricism would not have been suitable choices in view of the inquiry question. The qualitative method, therefore, became the inevitable choice in this case.

### Thematic analysis

The choice of thematic analysis was prompted because it is generally perceived as the foundation method in qualitative research (37). The combination of procedural simplicity with a technical background gives thematic analysis an edge over other qualitative analysis methods, thus making it one of the most widely used qualitative methods. Furthermore, it not only gives a succinct description and interpretation of the themes and patterns from the data (38) but also has the advantage of providing flexibility to the researchers as it is not tied to a particular epistemological or theoretical perspective (39). Thematic analysis in a study can be embedded in an essentialist/realist, constructionist or contextualist method (37). The current study employed the constructionist method as it explored how the participants’ events, realities, meanings and experiences were influenced by their cultural and religious contexts (40).

### Data collection method

To gather details of the experiences of individuals regarding FNSD ethology, semi-structured in-depth interviews were conducted. Each participant was interviewed once. The interviews lasted for a duration of 2 h on average. The interviews were conducted conversationally in a quiet and comfortable room to let the participants share the cultural/religious ambivalence in their own lives.

### Sampling/participants

The sample consisted of 22 participants (5 men and 17 women) whose ages ranged between 20 and 35 years and they were all single. Regarding occupation, nine participants were doing government or private jobs, six were homemakers, five were students and two were businessmen. The participants were selected through purposive sampling (See Table 1 for demographics).

**Table 1:** The demographic characteristics of the participants.

Pseudonyms	Gender	Age range (years)	Education
Samina	Female	20–25	FA
Faiza	Female	20–25	BA
Umer	Male	25–30	MS
Maryam	Female	30–35	MPhil
Kashif	Male	20–25	FSc
Uzair	Male	20–25	FA
Amina	Female	20–25	4th year (University)
Asfa	Female	20–25	3rd year (University)
Shafiq	Male	35–40	BA
Maliha	Female	25–30	MSc
Saman	Female	25–30	BA
Saeeda	Female	30–35	MPhil
Bilal	Male	25–30	MA
Ayesha	Female	25–30	BA
Sadaf	Female	20–25	2nd year Medicine
Khola	Female	20–25	MSc
Tayyaba	Female	20–25	BSC (Hons)
Fariha	Female	25–30	MA
Rabia	Female	25–30	MA
Saba	Female	20–25	FA
Nabila	Female	25–30	BA
Sonia	Female	20–25	FA

### Inclusion criteria

In this study, we selected only those participants who fulfilled the diagnostic criteria for FNSD (with psychological stressors) according to DSM-V. As the interviews were conducted in Urdu, the national language thus, fluency in Urdu was a prerequisite for the participants. Interestingly, all the participants were single, which was not a part of the inclusion criteria and hence a mere coincidence. Lastly, the participants with a minimum of 12 years of education were included in the study to exhibit psychological sophistication and adequately answer the main inquiry question. Initially, 25 participants were selected, out of which two withdrew when the aims and objectives were explained to them as they did not wish to talk about cultural and religious conflicts and have it audiotaped, while one had to go out of the station on account of a close death in the family. Thus, the total sample consisted of 22 participants.

### Ethical considerations

The Departmental Doctoral Programme Committee approved the study at the Centre for Clinical Psychology, University of the Punjab, Lahore, Pakistan. The first two authors have been part time faculty members at this institute for the past many years, while the third author also served as a lecturer there for a year. Moreover, the first three authors are also alumni of the Centre for Clinical Psychology, University of the Punjab, Lahore. The participants were briefed about the nature and objectives of the research. Informed consent was taken from the participants, who were also briefed about their role in the research. Moreover, confidentiality was ensured, and they were told that they would be assigned fictitious names in the final write-up of the study. Lastly, the participants were informed that they would not be given any monetary incentive for the study. However, if the interview caused stress in them, they would be offered free therapy sessions at Services Hospital, Lahore, Pakistan.

### Procedure

Before conducting the interviews, an interview guide (see Table 2) was developed to help the interviewer get detailed information from the participants. At the time, the study was conducted the first author was working as a senior clinical psychologist at the Department of Psychiatry, Services Hospital, Lahore. A meeting was held with all the senior psychiatrists and the psychologists working in the department and they were requested to refer diagnosed patients of FNSD with psychological stressors to the first author, so that they could be screened for inclusion in the study. The total number of clinical psychologists working at the department was four. Although the first author was a part of the care team, she was not the therapist of the participants. All the interviews were conducted by the first author in a comfortable room of the department of psychiatry at Services Hospital, Lahore, Pakistan. The first 15 min were spent developing a rapport which helped to put the participants at ease. It was done by giving factual information regarding the interview process, actively listening, and using appropriate non-verbal communication (41). This paved the path toward unfolding the participants’ narratives in a detailed and comprehensive manner. Furthermore, all interviews were audiotaped with the participants’ permission, and the queries (if any) regarding the audio taping were answered. The central inquiry question was “What role did cultural/religious factors play in the development of your illness?” (See Table 2).

**Table 2:** Interview guide questions.

Sr. No.	Questions
1.	Tell us about the first time you experienced symptoms (of FNSD)?
1a.	What were the circumstances that happened prior to the episode?
2.	How did culture have an impact on your present psychiatric condition?
2a.	What role did religious values play in your illness?
2b.	Have you experienced any cultural or religious conflict in the recent past?
3.	What strategies do you use to deal with the cultural/religious conflicts?

### Data analyses and verification

Thematic analysis was carried out following the six steps outlined by Braun and Clarke (37). The data obtained through interviews were transcribed, and then the first step was to get familiar with the data by reading, re-reading and taking initial notes. Initial codes were generated in the second step, while the third step comprised of collating the codes into potential themes. These themes were reviewed and later refined and named in the fourth and fifth steps, respectively, and sub-themes were also derived. In the sixth and final step, effective extracts were selected and related to the research question and literature, and the results were formulated and reported. For peer review, the analysis was shared with two experts in the field of psychology and qualitative research. One was an assistant professor working in a government university with 12 years of teaching and research experience.

In comparison, the other peer researcher was the head of the department of a private university with 15 years of experience. They were provided with the transcripts and the extracted themes and subthemes as well as paper trail and memos and were asked to review and assess the thematic clusters derived from the transcripts. Peer researchers assessed whether any point needed to be added or eliminated (42). All the suggestions from the peers were considered, and relevant changes were made accordingly. The respondent validation check was done in two ways. Firstly, the participants were asked to review the transcripts and identify any inaccuracies between the data and their responses during the interview (43, 44). Later, they were asked to review the accuracy and relevance of interpretations made by the researchers from the transcripts (43, 44). The participants suggested minor changes, which were incorporated into the analyses.

The themes in thematic analysis can be theoretical or deductive, driven by the research question, or inductive, which may not display many relations to the specific questions asked and are strongly linked to the data (37, 45). While doing the analysis, we came across an amalgam of deductive and inductive themes. This was an advantage of using a flexible approach, i.e., thematic analysis. As this study was a preliminary attempt to address the ambivalence faced by individuals with FNSD, it seemed best to report whatever was reflected by the data. Our first theme was deductive or theoretical, while our second was inductive.

## Results

This section mainly focuses on emergent themes. After careful analyses of the 22 transcripts (see Table 2), two main themes were identified: cultural and religious values and beliefs about romantic relationships. These themes and their subthemes displayed a direct or indirect relationship in the onset of FNSD.

### Cultural and religious values

From the very beginning, people are told to behave in a certain way, and this pattern is widely expected in South Asia (46), in which they are given a set of rules or ways of life which they are anticipated to follow. These rules represent their familial, cultural and religious norms and values that guarantee acceptance in their community and ensure affiliation. Cultural and religious values emerged as a significant theme in this study, which displayed a clear connection between the rules set by society and the burden of following them to fit in. Often, practices such as how marriages are arranged, the proper way to show respect for elders, etc., are influenced by familial/cultural norms and the pressure to follow them.

This central theme, cultural and religious values, was further divided into three sub-themes: self-perception, sexual suppression and family environment.

#### Self-perception

It is imperative for individuals to develop a positive image of themselves to have stable emotional regulation (47, 48). It is believed that perception of one’s behavior is directly linked to emotional adaptation. Participants in this study presented themselves as very religious. Their self-concept reflected their idea of themselves as quite religious, although they did not always follow the daily rituals required by Islam, such as praying five times a day, fasting, etc. They perceived their thoughts and feelings to be aligned with religion; they deemed inner faith superior to the mere practice of rituals.

Samina considered herself religious and even in her dreams, she often saw herself reciting the Holy Quran or preaching. She did not practice daily rituals regularly as she considered human values like not hurting anyone to be superior to rituals:

Let me tell you one thing (chuckled), I don’t pray regularly, and often I feel guilty about it but I consider myself quite religious as I don’t hurt people. I have seen many people, they pray a lot but they hurt people with their words. I think it’s more spiritual to be kind to others.

Likewise, Faiza, also perceived herself very religious, said:

I am very religious and have a strong connection with God and feel very guilty if I deviate from this self-image.

For Samina and Faiza, it was more important to adhere to the ideals of Islam than to simply practice the rituals of daily prayers.

#### Conviction in religious beliefs

Muslims believe that to be a good Muslim, one must follow the tenets of Islam in their daily life, such as the five times prayers that are offered daily, the consumption of halal food, modesty in attire, etc. Some people incorporate religious rituals into their daily routines to such an extent that their whole life is governed by them. For example, for Umer, conducting his life strictly according to religion was a way of life. He considered listening to music or watching TV un-Islamic. He said:

I used to switch off the TV. I don’t even remember the images of television. I even ask for forgiveness from God for all the sins I have committed in the past like watching movies, dramas and listening to songs. The thought of punishment for my wrongdoings disturbs me to my core and I try to suppress them by praying.

For Maryam, religion effectively dealt with life stressors. Several months ago, at a family event, her aunt told all the present relatives that Maryam had abused her, which was not true. Moreover, she made Maryam apologize to her publicly for this false allegation. Maryam was quite distressed, but she resorted to religion to deal with her stress:

At first, I was really disturbed and started having fits (pseudo seizures). Then I left the matter to God and ultimately, I got over it and found solace. This strengthened my faith in the belief that all matters should be left to God. Consequently, my fits (pseudo seizures) also improved.

#### Sexual suppression

In various cultures/religions, shame is associated with sexual expression. Therefore, people often refrain from explicitly discussing sex, especially with the opposite gender. In this study, all the participants seemed to exhibit suppressed sexual expression and severe guilt. Bilal admitted having sexual thoughts and added: “I spend three days with a jammat (religious group) because it makes me get rid of sexual thoughts for the next 6–7 months.” Additionally, Kashif denied any sexual fantasies even though he had a romantic relationship for almost a year. He said:

I consider having sexual fantasies a sin and a punishment for myself. I don’t see women with a sexual perspective. I don’t know how I got involved, but even in this relationship, I have set a moral standard and never touched her. Whenever such shameful thoughts come to my mind, I get extremely disturbed and I tried to shrug the thoughts off by reciting Kalma (Islamic phrases recited by Muslims).

Likewise, Uzair had been involved in a romantic relationship for 2 years and shared:

Let me tell you an incident, one day I was talking to my girlfriend on the phone and the conversation became sexual. I knew it was my mistake so I got very angry at myself and couldn’t believe that I had stooped to that level. I thought that this was not me, this is not what my religious and cultural values teach me. The next morning, I couldn’t speak.

Umer, who considered pre-marital relationships haram (Religiously prohibited), mentioned:

One day when I opened my shop a girl came and asked for my phone number, my heart started beating very fast I swear I am not a bad guy.

### Beliefs about romantic relationships

**Figure 2:**
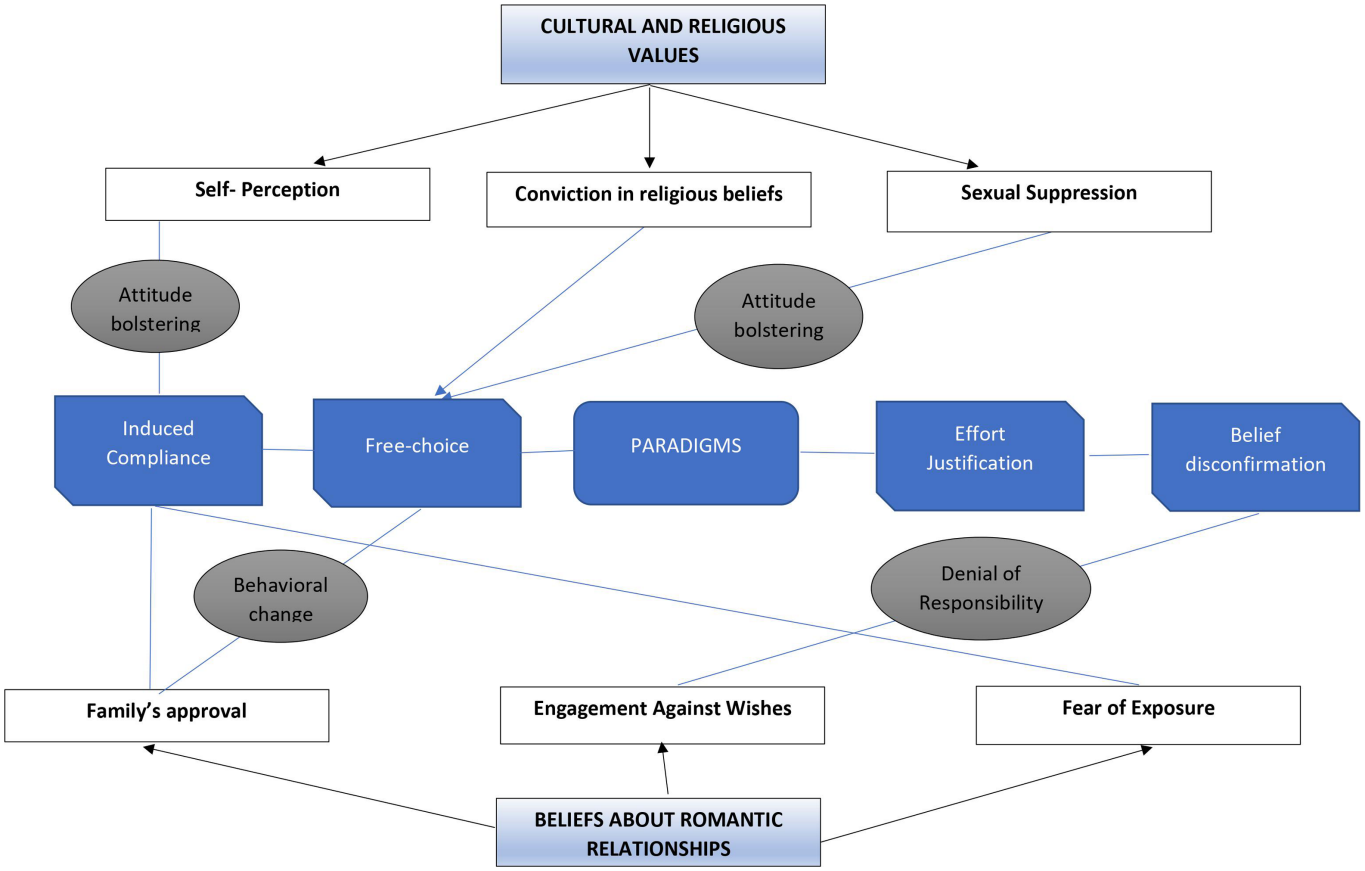
The paradigms from CDT in relation to the themes identified in the study. The bubbles in gray represent the reduction strategy used to manage the dissonance within each theme. For example, attitude bolstering was the reduction strategy found in subthemes like self-perception and sexual suppression. At the same time, behavioral change and denial of responsibility were the two reduction strategies used in two subthemes, i.e., family’s approval and engagement against wishes.

Every participant in the study was involved in romantic relationships. At the same time, they exhibited fear of disapproval by God, their families, and society around this involvement, which created a state of conflict in them. The predominant sub-themes are listed below (Figure 2):

#### Family’s approval

Some participants needed the approval of at least one family member as a “go-ahead” for their romantic involvement before committing. For instance, before sharing her feelings with the person she had fallen in love with, Amina shared her feelings with her mother, who disapproved of the match:

One day I shared with my mother that I really liked a cousin from her family, but she immediately brushed the idea aside saying that the two families had conflicts and would not approve of this marriage. So, I should forget about it and though it was tough, I complied but it caused anger and led to fits (pseudo seizures).

Similarly, Ayesha, Tayyaba, and Maliha had strong feelings for their partners. However, their families did not approve, and Sadaf felt guilty for falling in love because she knew that her family would never approve. In some cases, one of the parents may approve, but in Pakistan’s religious and cultural setup, collective family approval is often required. For instance, Asfa mentioned:

When I developed feelings for my cousin, I shared them with my mother and she reassured me that she would convince my father to get me married with him. So, I made a commitment with him but I stayed disturbed knowing how hurt my father would feel on discovering that I was involved with my cousin.

#### Engagement against wishes

Some participants got engaged against their wishes to please their families though they were in a relationship with another person then. For instance, Fariha and Rabia were engaged but expressed their displeasure around it; Fariha mentioned: “my engagement is my main stress, and I have never liked my fiancé, but I am helpless.” Furthermore, Shafiq added:

I knew that if I say “no” to my cousin’s marriage proposal it could lead to a lot of strain between two families. Even though I was in a relationship already, I decided to go along with the family decision for the time being and plan some strategy to break the engagement later. However, this created a lot of mental strain for me, because I knew that I was deceiving my family and couldn’t ditch my girlfriend as well. I was in a constant battle between my own desires and my family’s expectations of me.

Similarly, Asfa told:

I stayed quiet at the time of engagement as it would have created an uproar in the family. I wasn’t happy at all because I was in love with someone else, but I hoped that I would later share it with my family and make them understand. It was only an engagement, we were not legally married.

#### Fear of exposure

At times, the participants refrained from getting thoroughly involved in their relationship because they feared that people, especially their families, may find out about it, and this caused fear of dire consequences among them. Sonia explained her feelings and said:

I can’t imagine the humiliation my family would face if they get to know that I love him.

Moreover, Maliha shared:

Mustafa said that he wanted to marry me even if my family didn’t agree. He also said that he would ensure that I didn’t get married to anyone else. I was really stressed thinking that he may tell all these things to my future in-laws and tarnish my name. The thought of bringing shame to my family due to a boy disturbed me to my core and impacted my health badly.

Likewise, Saman said:

I did not want my father to think that I am immoral. I knew that there is no concept of “love marriages” in my family I was scared that if my father finds out about my relationship he may go to any extent because he would get really hurt and angry. I remained preoccupied and worried by these thoughts and later developed fits (pseudo seizures).

Family’s disapproval of involvement in a relationship before getting married instilled fear in Maliha and Saman. The fear was so intense that it distressed them immensely. While a significant number of people marry a person of their own choice, romantic relationships are generally frowned upon by society. Some people consider it unacceptable and a reflection of low moral values.

The two themes, i.e., cultural and religious values as well as beliefs about romantic relationships in this study indicate a state of conflict and dissonance that individuals residing in countries where religion is a strong institution, tend to experience. Pakistan was founded on religious principles resulting in a profound impact of religion on cultural and familial establishments, thus impacting various aspects of the individuals’ lives. An in-depth analysis of the accounts of the participants highlights a glaring conflict that they tend to experience between their personal desires and expectations from culture and religion. This is so because the way religion is mostly practiced at a preliminary level, and the emphasis is on unconditional surrender and conformity. Ironically, unconditional surrender can only come once an individual discovers and strengthens himself through love and spiritual closeness with God (49, 50). Therefore, the need to conform to the authorities of religion and culture in the absence of an acceptable rationale can cause dissonance and lead to FNSD.

The researchers followed a methodology reminiscent of Braun and Clarke’s (37) approach, whereby they conducted an analysis of the participants’ narratives and semantics to comprehend the substance they attributed to their experiences. Subsequently, the researchers delved beyond the narratives to uncover the implicit assumptions and interconnections underlying the participants’ statements, a procedure informed by the scholarly contributions of Smith and Osborn (51), Alase (52) as well as Finlay and Gough (53).

## Discussion

This study aimed to gain insights into how the participants experienced the cultural and religious conflicts in their lives and the meanings they attached to them, which were implicated in the etiology of FNSD.

The two main themes that emerged from this study are *religious and cultural values* and *beliefs about romantic relationships*. These two themes are interconnected; beliefs about romantic relationships were interpreted and experienced through the perspective of religion and culture. It was evident from the data that religion, Islam in this case, played a significant role in the belief system of the participants. Thus, it was essential to gain an understanding of the participants’ interpretations of Islamic injunctions embedded in the socio-cultural context on which this interpretation was founded. For most people in Pakistan, boundaries between religion, culture, family, and romance are intricately woven together (54, 55). Religious beliefs, or rather the interpretation of religious injunctions, dictate social and familial relationships and underlie most life decisions. In Pakistan, separating religion from culture is not simple, as religion is embedded in cultural practices and vice versa (56, 57).

### Cognitive dissonance paradigms

Festinger’s theory of cognitive dissonance best explained the perspectives of the participants regarding the dilemmas they encountered and the way they processed them. According to Festinger’s theory, the process of neutralizing dissonance and reaching consonance is possible through the use of four paradigms: induced compliance, free choice, effort-justification, and belief disconfirmation.

On analyzing the data through the lens of cognitive dissonance theory, it appears there were instances where individuals’ cognitions were not consonant with their prior beliefs. For example, two participants, Samina and Faiza, who had a strong religious inclination, experienced cognitive dissonance when they did not comply with religious practices. This contradiction between belief and behavior refers to the *induced compliance paradigm*. Similarly, Umer was a strict believer and considered having pre-marital relationships *Haram. He* faced indirect, *induced compliance* when a girl asked for his phone number as it threatened his belief.

Efrati (58), who studied sexual suppression among religious and secular individuals, found elevated sexual suppression in religious individuals and that this sexual suppression also led to distress. Sexual suppression based on cultural and religious beliefs gets in the way of romantic relationships, and if they are involved in a romantic relationship, they experience cognitive dissonance (59). The paradigm of *induced compliance* regarding thought suppression and sexual oppression was seen in Bilal and Uzair, *where they experienced denial and guilt regarding sexual thoughts.* Similarly, Saman and Sonia knew that love marriage was not allowed in their families; consequently, having a romantic relationship caused cognitive dissonance because it created a contradiction between their beliefs and their current actions. The above examples indicate *the induced compliance paradigm.*

*The free choice paradigm*, which is another form of cognitive dissonance, was experienced by the majority of the participants. One of the participants, Kashif, reported that although he had a romantic relationship with a woman, he did not engage in any sexual activity. Likewise, Amina chose to forget about her relationship because she belonged to a conservative family, and her mother did not allow her to marry her cousin. Similarly, Umer considered himself a strict Muslim; he believed that activities such as watching TV and listening to music were prohibited in Islam, so he decided to stop watching TV and listening to music.

The above examples display dissonance emanating from contradictory behaviors because these individuals had to choose difficult options (refrainment from sex, leaving a romantic relationship, abstinence from watching TV and listening to music). The options they chose resonated with their cultural and religious values. All three represent the *free-choice paradigm* of Festinger’s model.

Yeung et al. (60) reviewed the literature on trends in South Asian living arrangements. They found that most individuals (aged above 18) lived with their parents, and the concept of arranged marriage was still very prevalent in South Asia. Similarly, in this study, all the participants lived with their parents, and their parents made significant decisions. Another paradigm of dissonance, effort *justification,* was observed in Shafiq and Asfa, who were already in relationships but agreed to get engaged to a person of their parent’s choice to avoid uproar in their families. Similarly, Rabia and Fariha hated being engaged, but they complied with their families. Their involvement in an unpleasant activity (engagement against wishes) to achieve a pleasant outcome (use the strategy to convince the family later on or avoid uproar in the family) referred to *the effort justification paradigm*.

There were also examples when more than one paradigm was at play simultaneously. Rabia, Fariha, Shafiq and Asfa got engaged under pressure to avoid the turmoil in their families (Rabia and Fariha’s case) and planned the strategy to end the engagement later on (Shafiq and Asfa’s case) was *effort justification*. At the same time, they were indulging themselves in something contradictory to their beliefs, referred to as *the induced-compliance paradigm*.

### Reduction

Dissonance creates discomfort, which leads to a person’s attempt to reduce the discomfort and hence the dissonance. Likewise, the discomfort from dissonant situations motivates these individuals to *reduce* their cognitive dissonance. The maladaptive ways to achieve consonance between cognition and behavior were seen as a constant pattern in the participants. Kashif denied any sexual behavior toward his girlfriend and even denied having any sexual thoughts because such behaviors or thoughts were not accepted in his religion. He reduced his cognitive dissonance through the recitation of *kalma.*

Similarly, Samina confessed that she did not pray five times a day but was kind to other people. Adding another behavior or action to reduce dissonance refers to *attitude bolstering.* Adding another behavior instead of directly addressing the conflicting behavior can cause chronic mental health illnesses (61, 62). However, Bilal admitted to having sexual thoughts and mentioned that he spent a few days with an Islamic group to get rid of the sexual thoughts.

Amina was asked by her mother to forget about her intimate relationship, and she complied under family pressure. Likewise, Bilal spent 3 days with an Islamic group to eliminate his sexual thoughts. This type of reduction refers to behavioral change. This maladaptive *behavioral change* caused anger and eventually led to Amina’s pseudo-seizures.

*Denial of responsibility* was seen in Shafiq and Asfa, who got engaged to people chosen by their parents due to family pressure, even though Shafiq and Asfa were in love with someone else. In this particular case, cognitive dissonance was reduced temporarily and maladaptively because they planned to end the engagements later.

To summarize, this study sought to understand individuals diagnosed with Functional Neurological Symptom Disorder (FNSD) using the dissonance-reduction process of Festinger’s model. The participants displayed the following paradigms of *cognitive dissonance: induced compliance, free choice,* and *effort justification*.

Cognitive dissonance was reduced through *attitude bolstering, behavioral change, and denial of responsibility.* It was found that dissonance and maladaptive ways to *reduce* the dissonance mutually resulted in stress and poor mental health in individuals.

### Implications, limitations, and conclusion

This study highlighted significant factors contributing to the development of symptoms of FNSD. The two main interrelated themes were cultural and religious values and beliefs about romantic relationships. The results from these themes suggest that cognitive dissonance and maladaptive ways to reduce dissonance have adverse consequences in patients diagnosed with FNSD. This research has important implications in the formulation of therapeutic interventions for patients diagnosed with FNSD, particularly those located in South Asian countries and also those who have a solid religious leaning. Insights into patients’ cultural and religious values can help understand how patients make meaning of their experiences and how they associate them with developing symptoms of FNSD.

One limitation of this study was the sample size. The sample consisted of 22 participants (5 men and 17 women). In order to increase the generalizability, future studies should consider a larger sample and replication of the study with a variety of populations. This may also deepen our understanding of FNSD, its etiology and variations in different populations.

Since the respondents of this study were primarily women participants and few men, one possible implication for future research on the topic could be to study patriarchal structures which do not necessarily emanate from cultural and religious norms and how they affect the development of FNSD among women. Another dimension vital to understanding FNSD is how patriarchal norms of submissiveness in women and patterns of contradictory behaviors could make women more vulnerable to the development of FNSD. Moreover, future research could also focus on the general dearth of mental health education in South Asian countries coupled with the religio-cultural boundedness in these societies and how they implicate FNSD-related problems among the population.

## Data availability statement

The original contributions presented in the study are included in the article/Supplementary material, further inquiries can be directed to the corresponding author.

## Ethics statement

The Departmental Doctoral Programme Committee approved the study at the Centre for Clinical Psychology, University of the Punjab, Lahore, Pakistan. Informed consent was taken from the participants, who were also briefed about their role in the research.

## Author contributions

IB conceptualized the study, collected and analyzed the data, and finalized the manuscript. UF conceptualized the study and analyzed the data as well as did the write-up. KT and ZS conducted literature search and helped to finalize the manuscript. All authors contributed to the article and approved the submitted version.

## Conflict of interest

The authors declare that the research was conducted in the absence of any commercial or financial relationships that could be construed as a potential conflict of interest.

## Publisher’s note

All claims expressed in this article are solely those of the authors and do not necessarily represent those of their affiliated organizations, or those of the publisher, the editors and the reviewers. Any product that may be evaluated in this article, or claim that may be made by its manufacturer, is not guaranteed or endorsed by the publisher.

## References

[1] Ali S Jabeen S Pate RJ Shahid M Chinala S Nathani M. Conversion disorder-mind versus body: a review. Innov. Clin. Neurosci. (2015) 12:27–33. PMID: 26155375 PMC4479361

[2] Fobian AD Elliott L. A review of functional neurological symptom disorder etiology and the integrated etiological summary model. J. Psychiatry Neurosci. (2019) 44:8–18. doi: 10.1503/jpn.170190, PMID: 30565902 PMC6306282

[3] Scamvougeras A Howard A. Somatic symptom disorder, medically unexplained symptoms, somatoform disorders, functional neurological disorder: how DSM-5 got it wrong. Can. J. Psychiatry. (2020) 65:301–5. doi: 10.1177/0706743720912858, PMID: 32191123 PMC7265612

[4] American Psychiatric Association. Diagnostic and statistical manual of mental disorders. (5th ed., text rev.). ed. Virginia: American Psychiatric Association (2022).

[5] Bokharey IZ Fahim U Tahir K. Family conflicts are bitter splits that hurt: a qualitative inquiry toward understanding the impact of family issues in functional neurological symptom disorder. Front. Psychol. (2021) 12:1814. doi: 10.3389/fpsyg.2021.652917, PMID: 34108912 PMC8180856

[6] Sperry L ed. Mental health and mental disorders: an Encyclopaedia of conditions, treatments, and well-being [3 volumes]: An encyclopedia of conditions, treatments, and well-being. Santa Barbara, CA: ABC-CLIO (2015).

[7] Peeling J. L. Muzio M. R. (2020). Conversion disorder [Updated December 16, 2019] StatPearls [internet]. Treasure Island (FL): StatPearls Publishing. Phenomenological Analysis in qualitative research psychology. Czasopismo. Available at: https://www.ncbi.nlm.nih.gov/books/NBK551567/

[8] Kring A Johnson S Davison GC. Abnormal psychology. London: Wiley Global Education (2012).

[9] Ahmad QA Bokharey IZ. Resilience and coping strategies in the patients with conversion disorder and general medical conditions: a comparative study. Malays. J. Psychiatry. (2013) 22:39–50. Available at: https://www.semanticscholar.org/paper/Resilience-and-Coping-Strategies-in-the-Patients-A-Ahmad

[10] Bokharey IZ Rahman NK. Development of an indigenous symptom checklist for conversion disorder. Pak. J. Clin. Psychol. (2013) 12 Available at: https://pjcpku.com/index.php/pjcp/article/view/84

[11] Keller H Greenfield PM. History and future of development in cross-cultural psychology. J. Cross-Cult. Psychol. (2000) 31:52–62. doi: 10.1177/0022022100031001005

[12] Karim S Saeed K Rana MH Mubbashar MH Jenkins R. Pakistan mental health country profile. Int. Rev. Psychiatry. (2004) 16:83–92. doi: 10.1080/09540260310001635131, PMID: 15276941

[13] Cassum LA Cash K Qidwai W Vertejee S. Exploring the experiences of the older adults who are brought to live in shelter homes in Karachi, Pakistan: a qualitative study. BMC Geriatr. (2020) 20:1–12. doi: 10.1186/s12877-019-1376-8, PMID: 31906863 PMC6945572

[14] Smerecnik C Schaalma H Gerjo K Meijer S Poelman J. An exploratory study of Muslims adolescents’ views on sexuality: implications for sex education and prevention. BMC Public Health. (2010) 10:533. doi: 10.1186/1471-2458-10-, PMID: 20815921 PMC2940920

[15] Hofmann W. Nordgren L. (2015). The psychology of desire (pp. 244–245). New York City: The Guilford Press.

[16] Hollender MH. The case of Anna O: a reformulation. Am. J. Psychiatry. (1980) 137:797–800. doi: 10.1176/ajp.137.7.797, PMID: 6992602

[17] Tummala-Narra P. Cultural competence as a core emphasis of psychoanalytic psychotherapy. Psychoanal. Psychol. (2015) 32:275–92. doi: 10.1037/a0034041

[18] Festinger L. A theory of cognitive dissonance, vol. 2. Stanford, CA: Stanford University Press (1957).

[19] Anderton CL Pender DA Asner-Self KK. A review of the religious identity/sexual orientation identity conflict literature: revisiting Festinger's cognitive dissonance theory. J. LGBT Issues Couns. (2011) 5:259–81. doi: 10.1080/15538605.2011.632745

[20] Harmon-Jones E Mills J. An introduction to cognitive dissonance theory and an overview of current perspectives on the theory In: Harmon-Jones E, editor. Cognitive dissonance: Reexamining a pivotal theory in psychology. Washington, DC: American Psychological Association (2019). 3–24.

[21] Brehm JW. A brief history of dissonance theory. Soc. Personal. Psychol. Compass. (2007) 1:381–91. doi: 10.1111/j.1751-9004.2007.00035.x

[22] Maich KH. Reducing cognitive dissonance through effort justification: evidence from past studies and daily experience. Western Undergraduate Psychol. J. (2013) 1:1–6.

[23] Darrat A. A. (2016). Examining consumers' cognitive and behavioral responses to belief disconfirmation (Doctoral dissertation, Louisiana Tech University). Available at: https://digitalcommons.latech.edu/cgi/viewcontent.cgi?article=1093&context=dissertations

[24] Burns CP. Cognitive dissonance theory and the induced-compliance paradigm: concerns for teaching religious studies. Teach. Theol. Relig. (2006) 9:3–8. doi: 10.1111/j.1467-9647.2006.00255.x

[25] Voisin D Fointiat V. Reduction in cognitive dissonance according to normative standards in the induced compliance paradigm. Soc. Psychol. (2013) 44:191–5. doi: 10.1027/1864-9335/a000103

[26] Edelstein R Rosen Y. The Effect of the Induced Compliance Paradigm on Emotions During Inter-group Conflict. Peace Conflict Stud. (2015) 22:112–36. doi: 10.46743/1082-7307/2015.1283

[27] Stangor C. Changing attitudes by changing behavior. Principles of social psychology-1st international edition. Victoria, BC: BCcampus (2014).

[28] McGrath A. Dealing with dissonance: a review of cognitive dissonance reduction. Soc. Personal. Psychol. Compass. (2017) 11:e12362. doi: 10.1111/spc3.12362

[29] Cancino-Montecinos S Björklund F Lindholm T. A general model of dissonance reduction: unifying past accounts via an emotion regulation perspective. Front. Psychol. (2020) 3184:540081. doi: 10.3389/fpsyg.2020.540081 PMC7686841 33262719

[30] Beauvois JL Joule RV Brunetti F. Cognitive rationalization and act rationalization in an escalation of commitment. Basic Appl. Soc. Psychol. (1993) 14:1–17. doi: 10.1207/s15324834basp1401_1

[31] Sherman SJ Gorkin L. Attitude bolstering when behavior is inconsistent with central attitudes. J. Exp. Soc. Psychol. (1980) 16:388–403. doi: 10.1016/0022-1031(80)90030-X

[32] Festinger L Carlsmith JM. Cognitive consequences of forced compliance. J. Abnorm. Soc. Psychol. (1959) 58:203–10. doi: 10.1037/h0041593, PMID: 13640824

[33] Gosling P Denizeau M Oberlé D. Denial of responsibility: a new mode of dissonance reduction. J. Pers. Soc. Psychol. (2006) 90:722–33. doi: 10.1037/0022-3514.90.5.722, PMID: 16737370

[34] Hoshino-Browne E Zanna AS Spencer SJ Zanna MP Kitayama S Lackenbauer S. On the cultural guises of cognitive dissonance: the case of easterners and westerners. J. Pers. Soc. Psychol. (2005) 89:294–310. doi: 10.1037/0022-3514.89.3.294, PMID: 16248715

[35] Tao K Jin Y. Cultural impacts on cognitive dissonance and eWOM/eNWOM. Commun. IIMA. (2017) 15:4. doi: 10.58729/1941-6687.1369

[36] Cooper J. Cognitive dissonance: where we’ve been and where we’re going. Int. Rev. Soc. Psychol. (2019) 32:7. doi: 10.5334/irsp.277

[37] Braun V Clarke V. Using thematic analysis in psychology. Qual. Res. Psychol. (2006) 3:77–101. doi: 10.1191/1478088706qp063oa

[38] Majumdar A. Thematic analysis in qualitative research In: Khorsow-Pour M, editor. Research anthology on innovative research methodologies and utilization across multiple disciplines. Hershey, PA: IGI Global (2022). 604–22.

[39] Maguire M Delahunt B. Doing a thematic analysis: a practical, step-by-step guide for learning and teaching scholars. All Ireland J. Higher Educ. (2017) 9 Available at: http://ojs.aishe.org/index.php/aishe-j/article/view/335

[40] Wyczesany M Ligeza TS. Towards a constructionist approach to emotions: verification of the three-dimensional model of affect with EEG-independent component analysis. Exp. Brain Res. (2015) 233:723–33. doi: 10.1007/s00221-014-4149-9, PMID: 25424865 PMC4318980

[41] Kapur R. (2018). Research methodology: methods and strategies. Available at: https://cmapspublic2.ihmc.us/rid=1SG69305X-1FN0PGW-TK/Kapur%20-%20Research%20methodology%20Methods%20and%20strategies.pdf

[42] Guthrie KH. Contemplative qualitative inquiry: a review. Qual. Rep. (2020) 25:1736A–7A. doi: 10.46743/2160-3715/2020.4721

[43] Lincoln Y. S. Guba E. G. (1985). Naturalistic inquiry. London: Sage, 9, 438–439

[44] Torrance H. Triangulation, respondent validation, and democratic participation in mixed methods research. J. Mixed Methods Res. (2012) 6:111–23. doi: 10.1177/1558689812437185

[45] Assarroudi A Heshmati Nabavi F Armat MR Ebadi A Vaismoradi M. Directed qualitative content analysis: the description and elaboration of its underpinning methods and data analysis process. J. Res. Nurs. (2018) 23:42–55. doi: 10.1177/1744987117741667, PMID: 34394406 PMC7932246

[46] Croissant A Trinn C. Culture, Identity and conflict in Asia and Southeast Asia. Asien: the German journal on contemporary Asia. ASIEN. (2009) 110:13–43. doi: 10.11588/asien.2009.110.17488

[47] Ackerman PL. Intelligence-as-process, personality, interests, and intelligence-as-knowledge: a framework for adult intellectual development In: Flanagan DP McDonough EM, editors. Contemporary intellectual assessment: theories, tests, and issues. New York City: The Guilford Press (2018). 225–41.

[48] Heine SJ. Positive self-views: understanding universals and variability across cultures. J. Cult. Evol. Psychol. (2004) 2:109–22. doi: 10.1556/JCEP.2.2004.1-2.7

[49] Fuller A. R. (1994). Psychology and religious eight points of view. (3rd). pp. 134–216. Lanham, MD: Rowman and Littlefield Publishers.

[50] Iqbal AM. The reconstruction of religious thoughts in Islam. Lahore: Ashraf Printing Press (1982).

[51] Smith J Osborn M. Interpretative phenomenological analysis as a useful methodology for research on the lived experience of pain. Br. J. Pain. (2015) 9:41–2. doi: 10.1177/2049463714541642, PMID: 26516556 PMC4616994

[52] Alase A. The interpretative phenomenological analysis (IPA): a guide to a good qualitative research approach. Int. J. Educ. Liter. Stud. (2017) 5:9–19. doi: 10.7575/aiac.ijels.v.5n.2p.9

[53] Finlay L Gough B eds. Reflexivity: a practical guide for researchers in health and social sciences. Hoboken, NJ: John Wiley and Sons, Ltd (2008).

[54] Doron A Broom A eds. Health, culture and religion in South Asia: critical perspectives. London: Routledge (2013).

[55] Khera GS Ahluwalia MK. The cultural closet: the south Asian American experience of keeping romantic relationships secret. J. Multicult. Couns. Dev. (2021) 49:18–31. doi: 10.1002/jmcd.12203

[56] Metcalf BD ed. Islam in South Asia in practice (vol. 33). Princeton, NJ: Princeton University Press (2009).

[57] Zaidi AU Couture-Carron A Maticka-Tyndale E. ‘Should I or should I not’? An exploration of South Asian youth's resistance to cultural deviancy. Int. J. Adolesc. Youth. (2016) 21:232–51. doi: 10.1080/02673843.2013.836978

[58] Efrati Y. God, I can’t stop thinking about sex! The rebound effect in unsuccessful suppression of sexual thoughts among religious adolescents. J. Sex Res. (2019) 56:146–55. doi: 10.1080/00224499.2018.1461796 29702013

[59] Ozyegin G. Gender and sexuality in Muslim cultures. London: Routledge (2016).

[60] Yeung WJJ Desai S Jones GW. Families in southeast and South Asia. Annu. Rev. Sociol. (2018) 44:469–95. doi: 10.1146/annurev-soc-073117-041124

[61] Indregard AMR Knardahl S Nielsen MB. Emotional dissonance, mental health complaints, and sickness absence among health-and social workers. The moderating role of self-efficacy. Front. Psychol. (2018) 9:592. doi: 10.3389/fpsyg.2018.00592, PMID: 29740375 PMC5928644

[62] Yousaf O Gobet F. The emotional and attitudinal consequences of religious hypocrisy: experimental evidence using a cognitive dissonance paradigm. J. Soc. Psychol. (2013) 153:667–86. doi: 10.1080/00224545.2013.814620, PMID: 24236379

